# Dietary countermeasure mitigates simulated spaceflight-induced osteopenia in mice

**DOI:** 10.1038/s41598-020-63404-x

**Published:** 2020-04-16

**Authors:** Sonette Steczina, Candice G. T. Tahimic, Megan Pendleton, Ons M’Saad, Moniece Lowe, Joshua S. Alwood, Bernard P. Halloran, Ruth K. Globus, Ann-Sofie Schreurs

**Affiliations:** 1 Blue Marble Space Institute of Science, Seattle, WA 98154 USA; 2 Space Biosciences Division, NASA Ames Research Center, Moffett Field, CA 94035 USA; 3 KBR, Moffett Field, California USA; 4 Department of Mechanical Engineering, University of California, Berkeley, Berkeley, CA 94720 USA; 5 Space Life Sciences Training Program, NASA Ames Research Center, Moffett Field, CA 94035 USA; 6 Department of Medicine, University of California, San Francisco, San Francisco, CA 94143 USA; 7 Universities Space Research Association, Moffett Field, CA USA

**Keywords:** Pharmaceutics, Bone

## Abstract

Spaceflight is a unique environment that includes at least two factors which can negatively impact skeletal health: microgravity and ionizing radiation. We have previously shown that a diet supplemented with dried plum powder (DP) prevented radiation-induced bone loss in mice. In this study, we investigated the capacity of the DP diet to prevent bone loss in mice following exposure to simulated spaceflight, combining microgravity (by hindlimb unloading) and radiation exposure. The DP diet was effective at preventing most decrements in bone micro-architectural and mechanical properties due to hindlimb unloading alone and simulated spaceflight. Furthermore, we show that the DP diet can protect osteoprogenitors from impairments resulting from simulated microgravity. Based on our findings, a dietary supplementation with DP could be an effective countermeasure against the skeletal deficits observed in astronauts during spaceflight.

## Introduction

Alterations in the gravity vector and exposure to ionizing radiation can disrupt skeletal homeostasis in mice^1–3^. There are multiple stressors associated with spaceflight, including microgravity and radiation which are known to cause bone loss^4–6^. Decrements in bone mineral density (BMD) have been observed in astronauts from the Mir missions as well as missions to the International Space Station (ISS)^7–9^. While much research has focused on the detrimental effects of microgravity on skeletal tissue, less is known about the impact of spaceflight radiation. Crewed missions have, to this point, primarily remained within low-Earth orbit (LEO). While sources of ionizing space radiation within LEO include galactic cosmic radiation and charged particles from unpredictable solar particle events (SPE)^10,11^, the presence of the Earth’s magnetosphere reduces exposure to ionizing space radiation. Missions beyond LEO pose the greatest risk of radiation exposure and is of significant concern for crew health^12–14^. Spaceflight-relevant radiation includes a mix of low-linear energy transfer (LET) species such as protons and helium ions as well as high-LET species such as iron^15,16^. Beyond LEO, for example, astronauts may be exposed to up to 0.7 Sv of ionizing radiation^12,15,17^ during a multi-year mission to the Moon or Mars^14,15,18^.

On Earth, bone homeostasis is effectively maintained by the controlled remodeling activity of bone-forming osteoblasts and bone-resorbing osteoclasts. However, exposure to low-LET radiation (^137^Cs or X-ray, 1–2 Gy) leads to a transient increase in the number of osteoclasts, accompanied by an increase in trabecular separation (Tb.Sp) and decrease in trabecular thickness (Tb.Th), overall leading to a reduction in bone volume fraction (BV/TV)^19–22^. Together, this early increase in bone resorption and decrease in bone formation due to radiation exposure can result in a state of osteopenia, potentially leading to an increased risk of bone fracture^16,23,24^. A possible mechanism of action responsible for these changes in bone homeostasis is the generation of reactive oxygen species (ROS) due to radiation exposure^2,25^. Furthermore, reduction of antioxidant defense mechanisms^26,27^ and activation of pro-inflammatory cytokines^16,28^ have been associated with altered redox-balance due to radiation exposure. Together, these critical changes in cellular signaling lead to compromised bone strength and increased fracture risk^25,29^. Due to the involvement of redox-signaling in radiation-induced bone loss, antioxidants have been considered as candidate countermeasures to mitigate spaceflight-induced bone loss^30^. Proposed mechanisms by which antioxidants exert protective effects include the quenching of ROS via an increase in antioxidant activity and decreasing pro-inflammatory signaling cascades^29^.

Dried plum, *Prunus domestica L*., has been shown to have beneficial effects against bone loss^31^ (reviewed in Wallace *et al*. 2017). Dried plum’s positive effects on bone health markers have been investigated in multiple models of osteopenia, in both human clinical trials^32–36^ and rodent studies^37,38^. While the mechanism of action of dried plum is still unknown, it has been hypothesized that the high antioxidant capacity and high polyphenolic content of this fruit scavenges free-radicals as well as promotes bone formation and inhibits bone resorption^39–42^. Our lab has reported that mice fed a diet composed of Dried Plum (DP) prevented cancellous bone loss caused by ionizing radiation (IR), both low-LET such as gamma (^137^Cs) and a mixture of both low-LET and high-LET (sequential beam of proton, ^1^H and iron, ^56^Fe). The proposed mechanism for DP’s protective effect is via the prevention of radiation-induced increases in markers of bone resorption, inflammation and oxidative stress^43^.

Since the spaceflight environment includes exposure to both IR and microgravity, we sought to extend our hypothesis that DP could be a countermeasure for both radiation- and microgravity-induced bone loss. The current literature indicates that simulated microgravity and IR each have detrimental effects on cancellous bone structure, although there is no consensus on whether simulated microgravity and IR cause additive or synergistic effects when combined^20,44,45^. Thus, more studies are needed to determine whether combining simulated microgravity and IR leads to cumulative effects compared to simulated microgravity and IR alone. Hindlimb unloading (HU) is widely used to simulate the effects of the microgravity environment in rodents^46^. This model allows for a simulation of the cephalad fluid shift^47^ and removal of load-bearing forces typically experienced by the hindlimbs, which can lead to increased numbers of bone-resorbing osteoclasts and decreased numbers of bone-forming osteoblasts^48^. Together, these events result in bone loss as well as reduced bone mechanical properties in rodents^49–52^. In addition, exposure to microgravity in mice adversely affects osteoprogenitors^53,54^. The bone marrow niche contains osteoblast progenitors cells^4^ and it has been shown that exposure to microgravity alters the cells proliferation capacity and impairs their ability to produce extracellular matrix, in turn inhibiting the maturation of the bone matrix. Thus, the ability to maintain bone homeostasis is diminished^16,54–56^.

To address these knowledge gaps related to spaceflight-induced bone loss and candidate countermeasures, we sought to evaluate the potentially distinct effects of microgravity and ionizing radiation when applied independently, or in combination. For this study, we utilized the HU model and exposure to total body irradiation (^137^Cs gamma radiation, at 2 Gy dose) as analogs of weightlessness and radiation exposure, respectively. A relatively high dose of radiation (2 Gy) was chosen as a positive control dose to ensure bone loss in rodents and to allow for testing of DP as a countermeasure, as previously described^43^. We sought to determine whether DP diet prevents simulated microgravity-induced bone loss alone, and in combination with radiation, as compared to respective control diet controls. We also assessed the ability of DP to mitigate simulated weightlessness- and/or radiation-induced changes to the axial skeleton^57^, specifically the lumbar 4 (L4) vertebrae. We analyzed both cancellous and cortical bone microarchitecture as well as mechanical properties of skeletal tissue. Additionally, we determined whether the DP diet had the capacity to protect osteoprogenitors after exposure to simulated microgravity, an essential part in the healthy maintenance of skeletal tissue.

## Results

### Body weight

**Figure 1:**
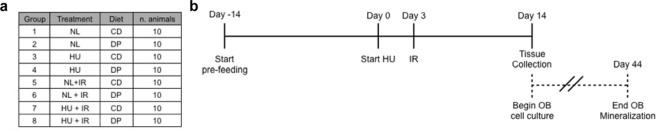
Experimental treatment groups and experiment design. (**a**) Male mice were assigned to groups (n = 10/group) and pre-fed with either a control diet (CD) or dried plum diet (DP). Mice were assigned to the following groups: 14 days of normally loaded conditions (NL) (*Groups 1,2*), hindlimb unloading (HU) alone (*Groups 3,4*), or HU in combination with ionizing radiation (IR), at a dose of 2 Gy ^137^Cs (0.8 Gy/min) (*Groups 5, 6, 7, 8*). (**b**) Mice were pre-fed with either CD or DP diets 14 days before onset of HU treatment, mice were exposed to IR 3 days after onset of HU, and samples were collected 14 days after initiation of HU treatment. At time of tissue collection (day 14), bone marrow cells were flushed from the femora’s of groups 1, 2, 3, 4 (n = 5/group) and cultured for 30 days for osteoblastogenesis (OB).

Skeletally mature, male mice, 14 weeks of age, were assigned to groups (n = 10/group) and pre-fed for 14 days with either a control diet (CD) or dried plum diet (DP) (Fig. 1). Mice were assigned to the following groups at the start of the experiment: normally loaded (NL) or hindlimb unloaded (HU) for a total of 14 days at 16 weeks of age, with either sham radiation or IR exposure on day 3. The experiment was completed on day 14 and samples of interest were collected (Fig. 1).

Body weights were recorded during the experiment to assess the overall health of the mice. As expected, HU mice showed decrements body weights starting from day 3 until day 9, at which point the body weights stabilize (Supplementary Table 1). In contrast, exposure to IR did not result in significant changes in body weights.

### Prevention of bone loss after exposure to simulated spaceflight

Micro-computed tomography (microCT) was utilized to quantify differences in bone microarchitecture after HU, IR or the combination of both treatments. We performed this analysis on both the cancellous and cortical regions of the appendicular (i.e. tibia) and axial (i.e. vertebra) skeleton.

#### Appendicular Skeleton

**Figure 2:**
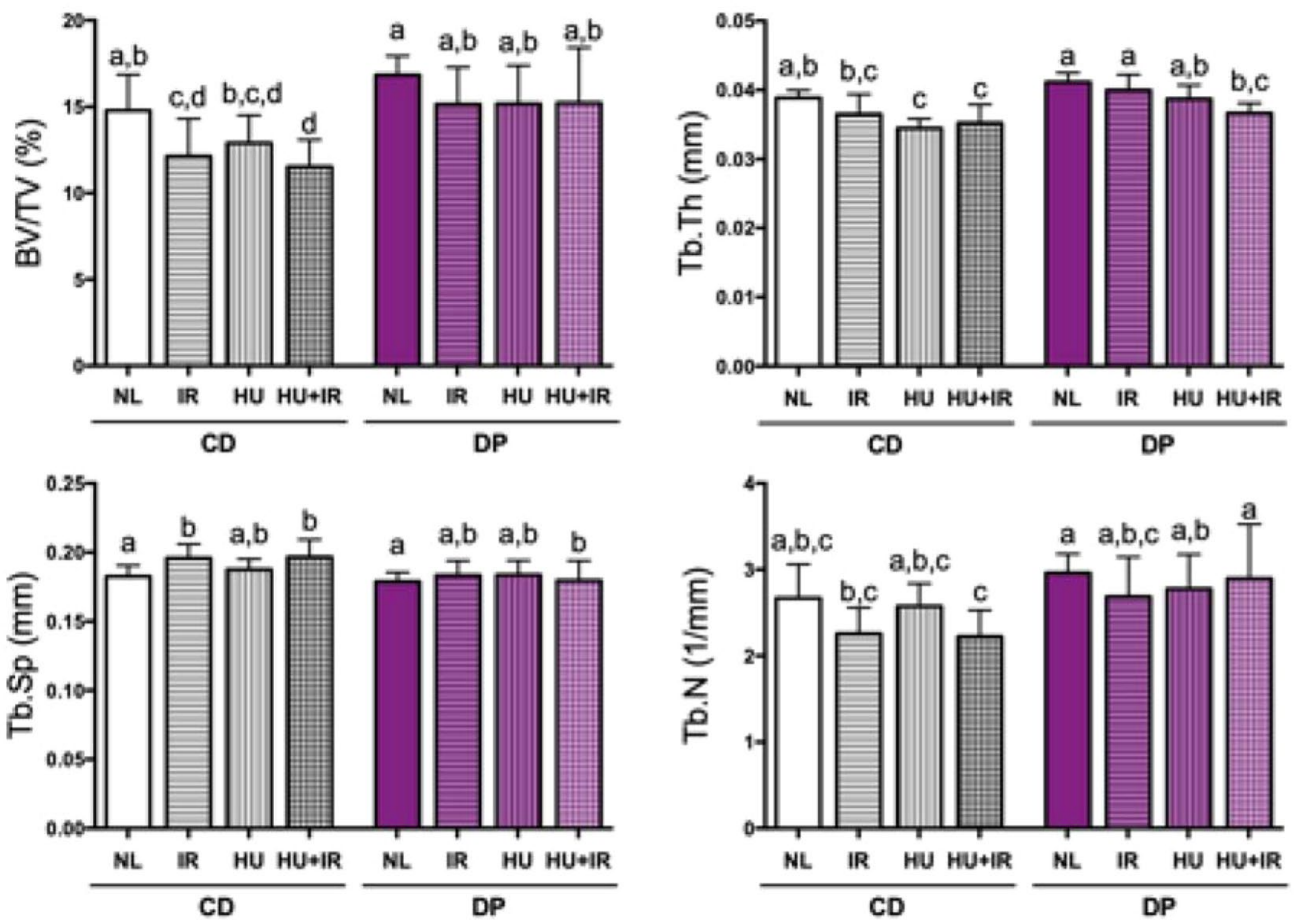
Effect of DP diet on cancellous bone loss induced by simulated spaceflight. Mice were separated into groups (n = 10/group), fed specific diets (either Control Diet, CD or Dried Plum diet, DP) and then were either unloaded to simulate weightlessness (HU), irradiated with 2 Gy ^137^Cs (IR), or a combination of the two (HU + IR). Tibiae were analyzed by microCT for cancellous bone parameters such as bone volume fraction (BV/TV, Panel a), trabecular thickness (Tb.Th, Panel b), trabecular separation (Tb.Sp, Panel c), and trabecular number (Tb.N, Panel d). Data shown are mean + /− S.D. Different letters indicate p < 0.05,1-factor ANOVA with all pairs Tukey Kramer.

**Figure 3:**
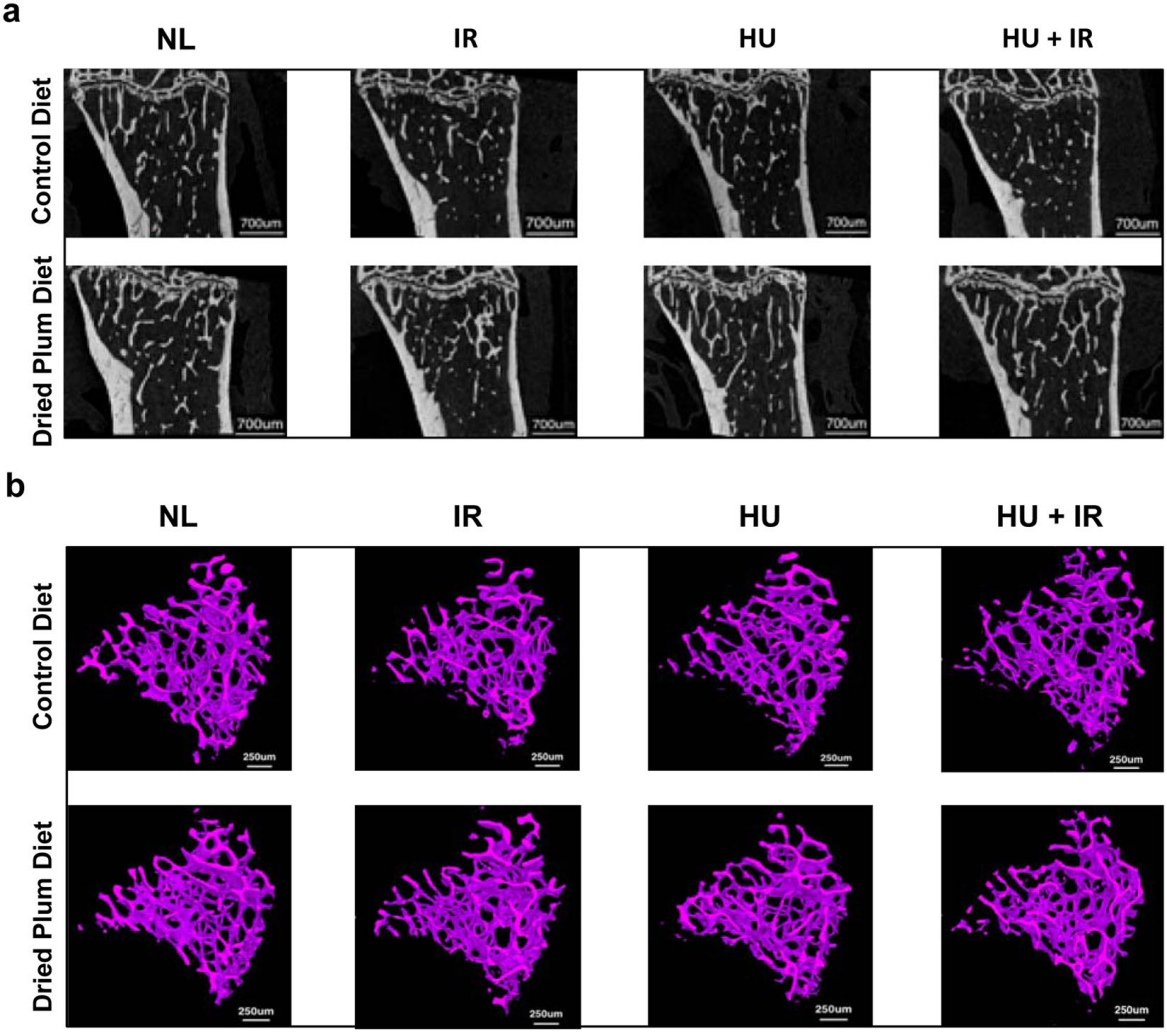
Coronal-slice microCT images and 3D reconstructions of cancellous bone of the tibia after exposure to simulated spaceflight. Mice were fed either CD or DP and then were either unloaded to simulate weightlessness (HU), irradiated with 2 Gy ^137^Cs (IR), or a combination of the two (HU + IR). Tibiae were imaged using microCT and 3D reconstructions were performed. (**a**) Representative coronal bone slices and (**b**) representative microCT images of cancellous bone after 3D reconstruction are illustrated for all treatment groups.

Mice exposed to simulated spaceflight factors, alone or combined, and fed a DP diet did not exhibit significant deficits in cancellous microarchitecture of the tibia compared to NL control mice, whereas mice fed the CD diet showed structural deficits in cancellous microarchitecture. In CD-fed mice, exposure to IR alone led to a 20% decrease in percent bone volume (BV/TV) and a modest but statistically significant 7% increase in trabecular separation (Tb.Sp) (Fig. 2, panels a and c), but did not decrease trabecular thickness (Tb.Th), nor trabecular numbers (Tb.N) compared to NL controls (Fig. 2, panels b and d). Similarly, exposure of CD-fed mice to HU alone resulted in an 11% decrease in Tb.Th (Fig. 2, panel b), but did not significantly affect the other parameters. In CD-fed mice, the combined treatment (HU and IR) led to a 25% decrease in BV/TV, accompanied by a modest but statistically significant 9% decrease in Tb.Th as well as an 8% increase in Tb.Sp when compared to NL controls (Fig. 2). Overall, mice fed the CD diet and exposed to simulated spaceflight led to a thinning of the trabeculae and greater separation between trabeculae, leading to a significant decrease in the BV/TV within the cancellous compartment. In DP-fed mice, the combination of HU and IR led to a statistically significant decline in Tb.Th relative to HU or IR alone. Together, these results indicate both a radioprotective effect of the DP diet on BV/TV and Tb.Sp, as well as a partial protection against HU-induced trabecular thinning. Of the cancellous parameters analyzed in the tibia, there were no significant differences due to diet alone between NL mice. Representative images are shown from each group in Fig. 3, with coronal view of the tibiae (panel a) and 3D reconstruction of the cancellous volume of interest (panel b), in which the skeletal decrements due to simulated spaceflight were observed in mice fed CD diet, but not in mice fed the DP diet.

To assess possible effects of a period of prefeeding mice with either CD or DP on microarchitecture, (and prior to initiating hindimb unloading), mice were prefed the diets for 17 days and the cancellous tibia analyzed by microCT. Mice fed the DP diet showed no effect of diet in BV/TV, Tb.N or Tb.Sp although a modest increase (+7%) in Tb.Th was observed (Supplementary table 2) which has been published before^35,37,58^.

To determine changes in cortical bone morphometry, two different locations of the tibia were analyzed, and the lengths of the tibiae were measured (Table 1). Tibiae lengths were unchanged after exposure to the treatments or diets. In CD-fed mice, cortical thickness at the proximal tibia was lower in mice exposed to HU (−10%) and the combination of HU and IR (−9%) compared to NL controls (Table 1). In DP-fed diet cortical thickness was lower only with combined treatment, not after exposure to HU alone when compared to the CD,NL group. No significant changes were observed in the cortical parameters measured at the distal tibia for mice fed either diet (Table 1).

**Table 1:** Cortical bone architecture of the tibia after exposure to simulated spaceflight.

	Control Diet				Dried Plum Diet			
	NL	IR	HU	HU + IR	NL	IR	HU	HU + IR
**Tibia Cortical**								
Tibia length (mm)	10.74 ± 0.18	10.80 ± 0.21	10.80 ± 0.19	10.90 ± 0.21	10.97 ± 0.23	10.90 ± 0.19	10.99 ± 0.21	10.89 ± 0.13
**Proximal tibia**								
Cortical thickness (mm)	0.167 ± 0.006 (a,b)	0.160 ± 0.008 (b,c)	0.150 ± 0.008 (c)	0.152 ± 0.01 (c)	0.172 ± 0.004 (a)	0.169 ± 0.009 (a,b)	0.160 ± 0.008 (b,c)	0.156 ± 0.009 (c)
**Distal tibia**								
TMD (g/cm^3^)	1.47 ± 0.04	1.46 ± 0.04	1.47 ± 0.04	1.47 ± 0.04	1.48 ± 0.05	1.47 ± 0.03	1.47 ± 0.04	1.47 ± 0.03
Periosteal perimeter (mm)	4.42 ± 0.26	4.30 ± 0.29	4.31 ± 0.17	4.41 ± 0.16	4.52 ± 0.18	4.36 ± 0.11	4.34 ± 0.23	4.38 ± 0.22
Endosteal perimeter (mm)	2.85 ± 0.26	2.83 ± 0.20	2.77 ± 0.11	2.84 ± 0.15	2.93 ± 0.14	2.82 ± 0.10	2.79 ± 0.14	2.85 ± 0.18
Cortical thickness (mm)	0.22 ± 0.01	0.21 ± 0.01	0.21 ± 0.01	0.22 ± 0.01	0.22 ± 0.01	0.21 ± 0.00	0.22 ± 0.01	0.21 ± 0.01
Moment of inertia (mm^4^)	0.21 ± 0.04	0.19 ± 0.04	0.19 ± 0.03	0.20 ± 0.02	0.22 ± 0.03	0.19 ± 0.02	0.20 ± 0.04	0.20 ± 0.04
Cortical bone volume (mm^3^)	0.21 ± 0.02	0.20 ± 0.02	0.20 ± 0.02	0.21 ± 0.01	0.22 ± 0.02	0.20 ± 0.01	0.21 ± 0.02	0.21 ± 0.02

Tibiae were analyzed by microCT for cortical bone parameters including periosteal perimeter, endosteal perimeter, cortical thickness, moment of inertia, cortical bone volume, and tissue mineral density (TMD). Data shown are mean + /− S.D. (n = 7–10/group). Different letters indicate p < 0.05,1-factor ANOVA with all pairs Tukey Kramer.

#### Axial Skeleton

**Figure 4:**
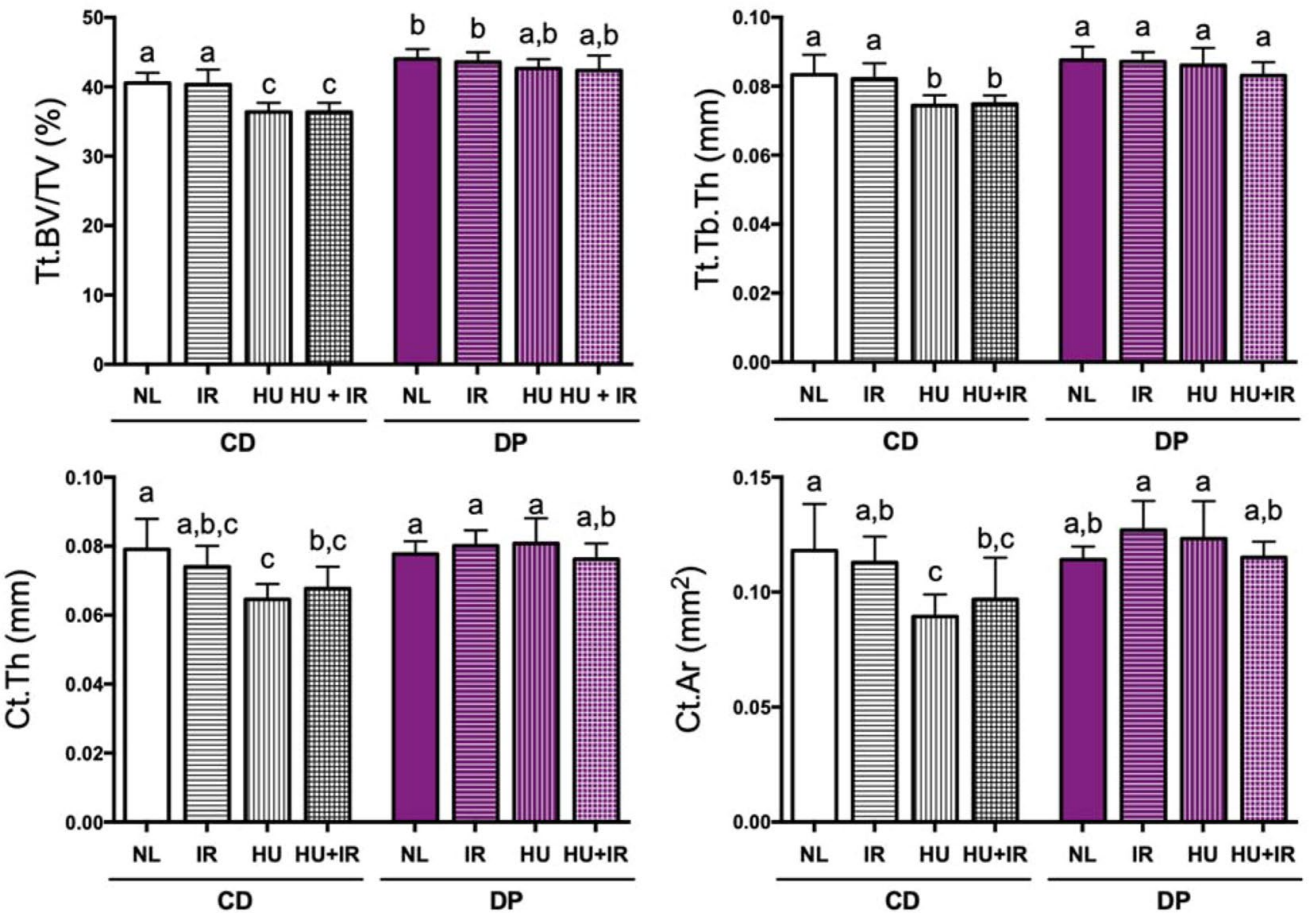
Cancellous and cortical bone loss of the vertebrae after exposure to simulated spaceflight. Mice were fed either CD or DP diets, and either unloaded to simulate weightlessness (HU), irradiated with 2 Gy ^137^Cs (IR), or a combination of the two treatments (HU + IR). Vertebrae were analyzed by microCT for changes in bone microarchitectural parameters such as total (Tt) vertebral body bone volume fraction (Tt.BV/TV, Panel a) and total vertebral body trabecular thickness (Tt.Tb.Th, Panel b) as well as for changes in cortical bone parameters including cortical thickness (Ct.Th, Panel c) and cortical bone area (Ct.Ar, panel d). Data shown are mean + /− S.D. (n = 7–10/group). Different letters indicate p < 0.05,1-factor ANOVA with all pairs Tukey Kramer.

Feeding mice the DP diet prevented bone loss in both cancellous and cortical compartments of the L4 vertebrae which was evident in CD-fed mice (Fig. 4). For vertebrae, the cortical shell and cancellous tissue were integrated to discern changes in total (Tt) vertebral body bone parameters. Whole vertebral body analysis revealed that CD-fed mice exposed to HU or HU + IR resulted in a 10% and 11% decrease in Tt.BV/TV, respectively (Fig. 4, panel a). Trabeculae showed reduced thickness after exposure to HU and HU + IR (−11% and −10%, respectively, Fig. 4, panel b), but Tb.Sp and Tb.N were not significantly affected (data not shown).

The posterior cortical shell of the vertebral body was computationally isolated from cancellous tissue to assess specific cortical parameters. Mice fed the CD diet showed decreased Ct.Th and Ct.Ar after exposure to HU (Ct.Th: −18% and Ct.Ar: −24%) and HU + IR (Ct.Th: −14% and Ct.Ar: −18%) (Fig. 4, panels c and d). In DP mice, the NL group had a 9% greater Tt.BV/TV compared to the CD, NL group, demonstrating an anabolic effect of the DP diet on cortical bone volume (Fig. 4, panel a). Mice fed the DP diet did not show any HU-induced decrements. Exposure to IR did not result in any changes to the microarchitecture of the vertebrae. No changes were observed in terms of vertebrae height (data not shown).

### Effects of the Dried Plum diet on vertebral strength

**Figure 5:**
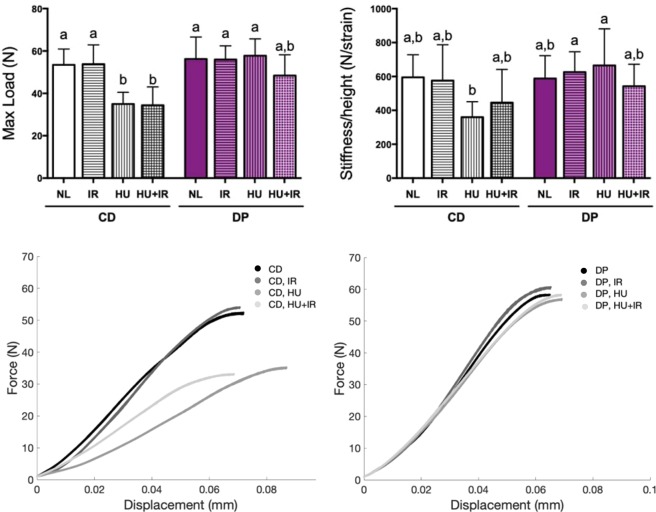
Differences in vertebrae compressive strength after exposure to simulated spaceflight. Mice were fed either the control diet (CD) or dried plum diet (DP), and were either irradiated with 2 Gy ^137^Cs (IR), hindlimb unloaded (HU), or treated with both (HU + IR). Monotonic compression testing was performed on the L4 vertebrae to obtain information about differences in maximum load (**a**) and vertebral stiffness (**b**). Stiffness was normalized to vertebral height. Representative force-displacement curves are shown for compression of the L4 vertebrae when mice were fed the CD diet (**c**) versus the DP diet (**d**). Data shown are mean + /− S.D. (n = 5–10/group). Different letters indicate p < 0.05,1-factor ANOVA with all pairs Tukey Kramer.

Monotonic compression testing of L4 vertebrae was performed to determine differences in mechanical properties of the bone tissue. Mechanical properties of interest included maximum load (i.e. the greatest force endured by the material before failure), and stiffness (i.e. the force needed to displace the material a standard distance). The maximum load for mice on the CD diet was 35% and 36% lower after exposure to HU and HU + IR respectively, compared to NL controls (Fig. 5, panel a). Based on our analysis, stiffness normalized to vertebral height in the CD fed groups did not meet criteria for a significant decrease when exposed to HU or HU + IR (Fig. 5, panel b). There was however a notable trend that HU (−40%) and HU + IR (−25%) led to decreased vertebral stiffness. We did however, see that there is an 85% greater stiffness in the DP fed, HU group compared to the CD-fed, HU group indicating that when exposed to HU, DP alone improved the stiffness of the vertebrae. Representative force-displacement curves are shown in Fig. 5 for the mice fed the CD diet (panel c) versus mice fed the DP diet (panel d). Mice fed the DP diet did not show significant decrements in maximum load or stiffness relative to CD-fed, NL mice. This suggests simulated microgravity reduced the strength of the vertebrae in CD-fed mice while mice fed the DP diet were protected against such deficits.

### Prevention of osteoblastogenesis impairment after exposure to hindlimb unloading

**Figure 6:**
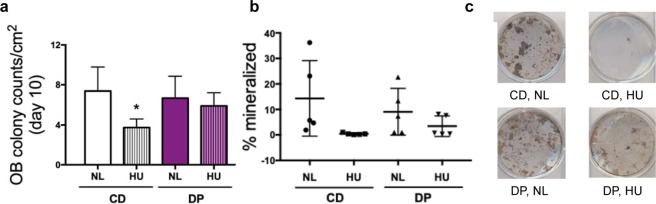
Simulated weightlessness induced decrement in e*x-vivo* osteoblastogenesis was prevented by DP. (**a**) Osteoblast colony counts at day 10 normalized to area of well (cm^2^). (**b**) Osteoblast percent mineralization at day 30. (**c**) Representative images of osteoblast mineralization at day 30 in the control diet (CD) and dried plum diet (DP) fed mice, after normally loading (NL) or hindlimb unloading (HU) for 14 days. Values are represented mean + /− S.D. (n = 5/group) and analyzed by 1-factor ANOVA for (**a**) and analyzed by nonparametric control Steel test for (**b**). *indicates p < 0.05 for appropriate statistical test.

Bone marrow cells were flushed from the femur and cultured with an osteoblastogenic media to differentiate the cells into bone-forming osteoblasts. Colonies formed by the osteoblasts in culture were counted on day 10 to quantify osteogenic cell growth and evaluated for osteogenic differentiation after 30 days in culture. Osteoblast mineralization was quantified as a percent area. Mice fed the CD diet had a significant decrease (49%) in colony counts (Fig. 6, panel a). CD-fed mice displayed a reduction in percent area mineralized after exposure to HU compared to the NL mice (97%). In contrast, mice fed the DP diet did not sustain any decrease in their osteoblast colony formation despite exposure to HU (Fig. 6, panel a). In addition, mice fed the DP diet did not show a significant decrement in percent mineralized area after exposure to HU (Fig. 6, panel b). Representative images of the mineralization are shown in Fig. 6, panel c. Taken together, the DP diet show a protective effect against HU-induced decrease in osteoblast cell proliferation and differentiation.

## Discussion

In this study, we investigated the bone protective potential of DP against independent and combined effects of simulated microgravity and ionizing radiation on the microarchitecture and mechanical properties of skeletal tissue. A diet supplemented with DP prevented most of the simulated spaceflight-induced damages to both the appendicular (i.e. tibia) and axial (i.e. vertebrae) skeleton. When mice were fed the control diet, a relatively high dose (2 Gy) of low-LET gamma radiation exclusively decreased the BV/TV and Tb.Sp of cancellous tissue in the tibia. Based on our results, cancellous bone loss was caused by a thinning, not a decrease in the number, of existing trabeculae, which overall, expanded the space between trabeculae. HU for 14 days caused bone loss within both cancellous and cortical regions of the tibia and the L4 vertebrae. HU also led to reduced compressive strength of the vertebral body. The independent effects of HU and IR remained of similar magnitude in each tissue compartment when mice were exposed to HU and IR simultaneously. Regardless of the treatment (with one exception, Ct.Th proximal tibia), consumption of the dried plum diet prevented detrimental skeletal changes.

Mice fed the control diet and exposed solely to IR displayed a 20% decrement in BV/TV and a 7% increase in the Tb.Sp of the tibia’s cancellous region relative to the sham control. These parameters were unaffected by HU alone. HU groups exhibited a lesser 11% decrement in Tb.Th. When IR was combined with HU, BV/TV decreased by another 5%. These results indicate that HU and IR were not clearly additive in this experiment. Among the parameters analyzed, IR only induced changes in Tb.Th and Tb.Sp. Unlike reports by others, there were no significant changes in Tb.N from any of the treatment groups relative to the sham-irradiated control group^21,43,44^. When HU was combined with IR, Tb.Th decreased by 9%.

Our results indicate bone loss was caused by a thinning, not a decrease in the number, of existing trabeculae, which expanded the space between trabeculae. The trabeculae contribute to percent bone volume and microarchitectural integrity. Since HU affects the cortical as well as cancellous tissue, we also determined the extent of cortical bone loss. The cortical region (cortical shell) exhibited a decrease in cortical thickness when mice fed the control diet were exposed to HU, either independently or in combination with IR.

When mice were fed the DP diet, IR-induced cancellous bone loss was entirely prevented, consistent with the radio-protective results reported in our previous study^43^. DP also protected the tibia when IR was combined with HU, which is a novel finding in this report. The decrease in trabecular thickness incurred by HU and HU + IR was entirely prevented by consumption of the DP diet. The DP diet also protected from HU-induced decrease in cortical thickness. Unlike the proximal tibia, the distal tibia did not show changes in cortical structure. In contrast, in the proximal tibia HU and IR together, not each treatment alone, caused a decrease in cortical thickness of the proximal tibia. This finding suggests that DP diet cannot fully prevent from all aspects of bone structural deficits induced by simulated spaceflight.

In order to confirm our results, we examined another skeletal site, the vertebra, which is a representative axial bone. Under conditions of simulated weightlessness and consumption of control diet, BV/TV and Tt.Tb.Th of the vertebral body were reduced accompanied by a reduction in cortical thickness and cortical bone area. Collectively, these findings indicate an overall deterioration of bone via thinning of the vertebrae’s trabeculae and cortical tissue. This deterioration of the microarchitecture directly impacted the overall strength of the tissue^57,59,60^, as reflected in the decrease in maximum load tolerable by the vertebral body, as well as the decrease in material stiffness as measured by mechanical compression testing. Taken together, these changes in both microarchitecture and strength have the potential to lead to fracture. However, these changes were not observed when mice were exposed to IR alone. In contrast to other studies where high-LET ^56^Fe radiation was utilized in combination with HU, our results did not indicate an additive effect of IR on the deterioration of strength and structure of the L4 vertebrae^57^. HU-induced damage to the vertebral body was entirely prevented when mice consumed the DP diet. Interestingly, there was a statistically significant increase in Tt.BV/TV (9%, Fig. 4, panel a) for sham-irradiated, NL mice fed the DP compared to the sham-irradiated, NL mice fed the CD. This elevated Tt.BV/TV was consistent for all treatment groups fed DP when compared to NL, CD-fed mice, suggesting a potential anabolic effect of DP.

Treatments that are currently in use to mitigate the effects of mechanical unloading are not without limitations and risks. Exercise in combination with drug treatments such as bisphosphonates have shown beneficial effects in astronauts^61,62^. However, in patients with osteoporosis, the use of bisphosphonates can increase the risk for atypical femoral fractures possibly due to suppressed bone turnover which may lead to cracks at the microscale and loss of mechanical strength^63,64^. Although rare, other notable side effects that have been reported to accompany bisphosphonate therapy including osteonecrosis of the jaw^65^ and atrial fibrillation^66^. Another limitation of bisphosphonates is that they act mostly on osteoclasts^61^.

Osteoblastogenesis from flushed bone marrow cells of the femur was strongly inhibited by exposure to HU, indicating that HU directly damages osteoblast progenitors, potentially affecting *in situ* bone formation (Fig. 6). Mineralization of the osteoblast cells from DP-fed mice was partially restored and taken together with the cell growth data, indicates that DP could prevent the loss of structure and strength by protecting the marrow-derived osteoprogenitors. *Ex vivo* osteoblastogenesis was only performed on mice exposed to HU because it has been shown previously that low-LET *in vivo* gamma radiation does not negatively impact osteoblasts^57,67^.

These data points toward dried plum playing an active role in protecting osteoprogenitors, possibly by maintaining the osteoblast’s growth and differentiation. Recently, Graef *et al*.^42^ showed that treatment of bone marrow-derived osteoblast cells with particular fractions of DP polyphenols led to an increase in gene expression of *Runx2* one hour after treatment as well as an increase in mineralized nodules^42^. When evaluated in combination with our findings, this supports the potential mechanism that dried plum activates osteoblasts, which may lead to an increase in bone formation. It should be noted, however, that another paper published by the same group showed that fractions of dried plum polyphenols were not as effective at preventing bone loss in a rodent model of ovariectomy-induced osteoporosis in comparison to a diet supplemented with whole dried plum^41^. Thus, follow-up studies are needed to compare the potential protective effect of these polyphenol fractions on bone exposed to simulated microgravity and ionizing radiation. In addition, establishing the efficacy of formulations of DP (dried whole fruit vs defined components) may facilitate the further development of DP as a countermeasure for spaceflight.

One limitation in our study is the lack of data on osteoclasts, and therefore we cannot determine if bone resorbing osteoclasts also contribute to the observed protective effects of the DP diet. Since HU and radiation can each increase osteoclasts, and a stimulation of bone resorption may contribute to the observed bone loss, DP may exert protective effects by inhibiting resorption, increasing formation, or both.

Overall, the ability of DP to protect osteoblast progenitor cells from HU-induced damage, as shown in this study, holds much promise for development of next generation anti-osteoporotic drugs due to the possibility that DP to act on both osteoblasts and osteoclasts. Our current study is limited to short-duration HU (2 weeks); since astronauts on long-term space missions may require a countermeasure for bone loss throughout the entirety of a multi-year mission beyond LEO, it is important in future studies to determine if DP is protective for long-term exposure to simulated, or actual, spaceflight. Further studies also are needed to gain more insight into any potential long-term side effects of consuming a dried plum diet. Dried plum’s potential as a countermeasure against both radiation- and microgravity-induced osteopenia such as loss of bone strength and structure in the tibia and vertebrae has important implications for astronauts in space as well as radiation workers, radiotherapy patients, and individuals with osteoporosis.

## Methods

### Animals and treatments

Male C57BL/6 J mice (Jackson Labs, Sacramento, CA) at 14 weeks of age were weight matched, assigned to groups (n = 10/group), and individually housed in standard vivarium cages or custom-designed HU cages as previously described^46^. Our justification for using mice of this age is that they are considered skeletally mature at the time of initiating treatment (HU and/or IR) so that the effects of HU and/or IR on the 16 week old mice can be attributed to dietary effects on the adult, rather than rather than on the growing skeleton. We have extensive previous reports showing this age and duration of HU and IR treatment yields the predicted bone loss (^45,57,68–70^). 14 week old mice were used to allow for a 2 week pre-feeding period. Two weeks of prefeeding period was selected to provide sufficient time for animals to adapt to the diet prior to initiating exposure to simulated spaceflight, while avoiding a lengthy pre-feeding period, which may potentially affect bone structural and/or mechanical properties prior to initiating treatments.

Animals assigned to the HU group were suspended with traction tape based on previously established procedures^46,71^. The room conditions was maintained at an average 73.3°F, 31% humidity, 12 hours light/dark cycle), with food and water provided *ad libitum*. Animal weights (Supplementary table 1) were monitored throughout the duration of the experiment to asses health of the mice. The NASA Ames Research Center Institutional Animal Care and Use Committee (IACUC) approved all procedures. All experiments were performed in accordance with relevant guidelines and regulations.

### Diets

Two diets were used for this study: The control diet (CD) was a defined formulation (AIN93M, Teklad, Madison, WI) while the DP diet was composed of the AIN93M diet supplemented with 25% by weight dried plum powder (from the California Dried Plum Board) and was compounded by Teklad as described previously^35,37,38,43,58,72,73^.

### Experimental protocol

Animals were pre-fed with either CD or DP diet for a period of 14 days in standard vivarium cages prior to undergoing sham-unloading or HU. Animals undergoing HU were allowed to acclimate to their new cage environment three days prior to initiating HU. Initiation of HU was considered to be day 0 of the experiment (Fig. 1, panel b). Sham-unloaded animals were handled similarly to HU mice, without undergoing actual tail suspension, followed by housing in NL conditions in vivarium cages. On day 3, animals were either exposed to sham-irradiation or total body IR. Mice were fed their respective diets until time of euthanasia (day 14). At time of euthanasia, tibiae, femora, and vertebrae were collected and processed for micro-computed tomography (microCT) or *ex vivo* cell culture.

### Radiation exposure

At 16 weeks of age, mice were exposed to 2 Gy Gamma of whole-body IR (^137^Cs at 0.83 Gy/min, JL Shepherd Mark I, San Gabriel, CA) as used in Schreurs *et al*.^43^. For this treatment, mice were housed individually in a custom animal holding cage, maintaining HU mice in a head-down position. Mice were placed in the irradiator chamber and exposed to TBI. The unloading of mice was maintained during irradiation by custom small HU cages. Sham-irradiated controls were handled similarly and placed in the irradiation chamber with the radiation source turned off.

### MicroCT

At the time of sample collection, tibiae were cut distal to the tibio-fibular junction (TFJ) to allow complete infiltration of fixative (4% paraformaldehyde, Sigma). After 24 hours at 4 °C, samples were transferred to 70% ethanol for long-term storage. The day before imaging, bones were rehydrated in phosphate buffered saline (PBS, Gibco 1×, pH 7.4). MicroCT was then conducted using SkyScan 1272 (Bruker, Kontich, Belgium), with a 0.25 mm aluminum filter, x-ray tube potential of 60 kV and x-ray intensity of 166 μA.

For the analysis of cancellous bone, scans were performed with a resolution of 3.5 μm/voxel resolution (integration time = 840 ms, frame averaging = 3, rotation step = 0.600°). A 1.0mm-thick region of cancellous bone located 0.5 mm distal to the proximal growth plate of the tibia was selected and auto-contoured as detailed by Buie *et al*.^74^, with minor modifications (SkyScan, CT Analyzer). From this auto-contouring program, bone volume fraction (BV/TV, %), trabecular thickness (Tb.Th, mm), trabecular number (Tb.N, 1/mm), and trabecular separation (Tb.Sp, mm) were calculated to assess bone microarchitecture and reported following conventional guidelines^75^. An automated contouring program was used to select and measure the microarchitecture of the cortical shell surrounding the abovementioned cancellous region. From this auto-contouring program, cortical thickness (Ct.Th, mm) and cortical bone area (Ct.Ar, mm^2^) were calculated.

To assess microarchitecture of the cortical compartment of the distal tibia, bones were scanned with a resolution of 12μm/voxel (integration time = 420 ms, frame averaging = 3, rotation step = 0.400°), and a 0.3 mm thick region located 2 mm proximal to the TFJ was selected and auto-contoured. Cortical parameters of interest included periosteal (Peri.P,mm) and endosteal (Endo.P, mm) perimeters, cortical thickness (Ct.Th, mm), mean polar moment of inertia (*J*, mm^4^), cortical bone volume (BV, mm^3^), marrow volume (mm^3^), marrow area (mm^2^), and volumetric tissue mineral density (TMD, g/cm^3^). Calcium hydroxyapatite phantoms (Bruker, Kontich, Belgium) were utilized to validate TMD by converting linear-attenuation coefficient of the material to TMD of the phantom^75^.

MicroCT analysis was also performed on the fourth lumbar (L4) vertebrae. Before scanning, vertebrae were processed to remove all transverse processes that project posteriorly in preparation for mechanical testing, as detailed below^76^. If a vertebral body was at all compromised during sample preparation, sample was removed from further analysis. Vertebrae were then scanned at 6μm/voxel resolution (integration time = 840 ms, frame averaging = 2, rotation step = 0.600°). Height of the vertebrae, in millimeters, was determined using the reconstructed scans. Analysis was performed on the entire, post-processed vertebral body^77^. An auto-contouring program was utilized to determine total (Tt)^78^ vertebral body bone volume fraction (Tt.BV/TV, %), total vertebral body trabecular thickness (Tt.Tb.Th, mm), total vertebral body trabecular number (Tt.Tb.N, 1/mm), and total vertebral body trabecular separation (Tt.Tb.Sp, mm). Total vertebral body is a measure of the microarchitecture of both the cortical and cancellous bone compartments. The geometry of the cortical shell was assessed separately at the posterior side of the vertebral body, as this region displays concentrated stress during uniaxial compression^57^.

### Mechanical testing – monotonic compression

The vertebral column was processed to isolate the L4 vertebra and further prepared for mechanical testing as described previously^76^. In brief, all soft tissue was carefully removed, and endplates and posterior elements were removed using a diamond-saw microtome (Leica SP1600 Microtome, Wetzlar, Germany) to isolate the vertebral body and ensure plano-parallel testing surfaces. Uniaxial compressive monotonic testing was performed as described previously^76^. In brief, after microCT was performed as detailed above, uniaxial compressive testing was performed at ambient temperature at a platen displacement rate of 0.01 mm/sec. Force (N) and displacement (mm) data were collected during testing (1,000 Hz; WinTest 7, version 7.01). Mechanical testing was not performed on samples in which the vertebral body was compromised during sample preparation. Thus, the sample size per group was as follows: n = 10 (CD, NL), n = 5 (CD, IR), n = 8 (CD, HU), n = 7 (CD, HU + IR), n = 8 (DP, NL), n = 6 (DP, IR), n = 9 (DP, HU), and n = 6 (DP, HU + IR). Custom MATLAB code (Version R2017a, The Mathworks, Inc., Natick, MA) was used to analyze the data and obtain maximum load at failure (N) and stiffness (N/mm). Stiffness is defined as the force required to displace a specimen a set distance, or the slope of the specimen’s force-displacement curve. Thus, stiffness was calculated as the slope of a line that best fit the middle 80% of data points in the range between the force at displacement = 0 and maximum load (i.e. a consistently linear region of the curve for all specimens). Reported stiffness (N/strain) was normalized by the height of the vertebra, as determined by microCT (Fig. 5, panel b). Due to the variation in cross-sectional area of the vertebral body from cranial to caudal ends, the elastic modulus and ultimate stress were not calculated.

### *Ex vivo* osteoblastogenic cell culture

Bone marrow cells were flushed and collected from the femur at time of euthanasia (n = 5 each for NL and HU groups). The limited number of samples per group were the result of the experimental design. Bone marrow cells were then plated at a density of 300,000 cells/cm^2^ in osteogenic media (α-MEM, FBS, Ascorbic Acid and beta-glycerophosphate), as described previously^2^. Media was changed every 2–3 days. On day 10 in culture, colonies consisting of at least 30 cells were scored. Colony counts were normalized to area of culture plate (3.8 cm^2^). Cells were grown for a total of 30 days to allow for mineralization, which was then stained using the Von Kossa method^2^ and quantified using ImageJ (Version 1.52a, NIH, USA).

### Statistical analysis

Data were presented as mean and standard deviations (S.D.). If the variances between groups were equal and parametric, a one-way analysis of variance (ANOVA) analysis was performed. When a main effect by one-way ANOVA (p < 0.05) was evident, an all pairs Tukey Kramers post-hoc analysis was performed (software JMP Version 13.0). We selected this formalism to allow for multiple and direct comparisons within each diet (CD or DP) and also across the diets (i.e CD HU + IR vs DP HU + IR) to assess our specific research objective, namely determining the efficacy of dried plum as a countermeasure of bone loss against the age-matched negative control. If the data were non-parametric, Steel or Wilcoxon post-hoc analysis was performed as needed.

## Supplementary information

Supplementary Table 1 and 2.
