# Supplementary material for: Dietary countermeasure mitigates simulated spaceflight-induced osteopenia in mice
Source: Sci Rep. 2020 Apr 16;10:6484. doi: 10.1038/s41598-020-63404-x (PMC7162976; doi:10.1038/s41598-020-63404-x)
Supplement: Supplementary file 1 — Supplementary Table 1 and 2. [file 41598_2020_63404_MOESM1_ESM.pdf]

Dietary countermeasure mitigates simulated spaceflight-induced osteopenia in mice

Sonette Steczina[1,2], Candice G.T. Tahimic[2,3], Megan Pendleton[4], Ons M'Saad[5], Moniece Lowe[1,2], Joshua S. Alwood[2], Bernard P. Halloran[6], Ruth K. Globus[2], Ann-Sofie Schreurs[2,7,*]

[1]Blue Marble Space Institute of Science, Seattle, WA 98154, USA
[2]Space Biosciences Division, NASA Ames Research Center, Moffett Field, CA 94035, USA
[3]KBR, Moffett Field, California, USA
[4]Department of Mechanical Engineering, University of California, Berkeley, Berkeley, CA 94720, USA
[5]Space Life Sciences Training Program, NASA Ames Research Center, Moffett Field, CA 94035, USA
[6]Department of Medicine, University of California, San Francisco, San Francisco, CA 94143, USA
[7]Universities Space Research Association, Moffett Field, CA, USA

*Corresponding author and material requests. NASA Ames Research Center, Moffett Field, CA 94035, USA. Email: ann-sofie.schreurs@nasa.gov.

	Average Body Weights						
	Day -14	Day -7	Day 0	Day 3	Day 6	Day 9	Day 14
Control Diet							
NL	28.17 ± 2.46	27.67 ± 2.47	27.77 ± 2.77	28.81 ± 3.24	29.96 ± 3.34	30.73 ± 3.57	30.79 ± 3.60
NL + IR	28.62 ± 2.29	28.68 ± 2.74	28.69 ± 2.63	29.67 ± 2.94	29.01 ± 2.47	29.27 ± 2.470	30.19 ± 2.70
HU	27.80 ± 0.79	27.40 ± 0.66	27.89 ± 0.52	26.69 ± 0.70	26.27 ± 0.73	26.22 ± 0.82	26.23 ± 0.89
HU + IR	27.30 ± 0.43	27.56 ± 0.43	27.96 ± 1.14	26.93 ± 1.08	25.21 ± 1.11	25.61 ± 1.25	26.01 ± 1.05
Dried Plum Diet							
NL	28.79 ± 0.41	29.22 ± 0.63	29.24 ± 0.81	29.55 ± 1.06	29.85 ± 0.99	30.51 ± 0.95	30.16 ± 1.23
NL + IR	28.02 ± 1.55	29.08 ± 1.56	28.79 ± 1.50	29.15 ± 1.70	28.26 ± 1.51	28.54 ± 1.83	28.87 ± 1.79
HU	28.03 ± 1.52	28.10 ± 1.73	27.69 ± 1.43	26.70 ± 1.64	26.34 ± 1.36	26.26 ± 1.59	26.34 ± 1.46
HU + IR	27.85 ± 1.79	28.22 ± 1.71	27.65 ± 1.42	26.69 ± 1.09	25.62 ± 1.09	25.26 ± 1.09	25.67 ± 0.99

Supplementary Table 1. Average body weights during the experiment. Average body weights were measured throughout the duration of the experiment for all treatment groups. Data shown are mean +/- S.D. (n=10/group).

	CD	DP	T-test
	NL	NL	
**Tibia Baseline Cancellous**			
BV/TV (%)	24.7 ± 3.4	26.5 ± 4.5	p = 0.4
Tb.Th (mm)	0.061 ± 0.0007	0.065 ± 0.0044	p = 0.04*
Tb.Sp (mm)	0.149 ± 0.017	0.151 ± 0.018	p = 0.7
Tb. N (1/mm)	4 ± 0.56	4.1 ± 0.55	p = 0.95

Supplementary Table 2. Baseline cancellous tibial microCT after 17 days of prefeeding. Mice were separated into groups (n=7-8/group) and fed specific diets for 17 days (either Control Diet or Dried Plum diet) while housed in standard cages. Tibiae were analyzed by microCT for cancellous bone parameters such as bone volume fraction (BV/TV), trabecular thickness (Tb.Th), trabecular separation (Tb.Sp), and trabecular number (Tb.N). Data shown are mean +/- S.D. *indicates $p<0.05$ by student t-test.
